# Effect of different intensity exercises intervention on cardiovascular functions and quality of life on patients with chronic heart failure: A protocol for systematic review and meta-analysis

**DOI:** 10.1097/MD.0000000000028554

**Published:** 2022-01-14

**Authors:** Yan Bai, Bin Hua, Fan Zhang, Wenqin Zhou, Bing Deng

**Affiliations:** a Department of Nursing, Longhua Hospital, Shanghai University of Traditional Chinese Medicine, Shanghai, China; b Department of Cardiology, Longhua Hospital, Shanghai University of Traditional Chinese Medicine, Shanghai, China; c Department of Nephrology, Longhua Hospital, Shanghai University of Traditional Chinese Medicine, Shanghai, China; d Nursing Teaching and Research Department, Longhua Hospital, Shanghai University of Traditional Chinese Medicine, Shanghai, China.

**Keywords:** chronic heart failure, exercise intensity, high-intensity exercise, meta-analysis, moderate-intensity exercise, protocol

## Abstract

**Background:**

Exercise training can improve exercise capacity, quality of life, and reduce hospitalization time in chronic heart failure (CHF) patients. Various training protocols have been studied in CHF, but there is no consensus on the optimal exercise intensity for the rehabilitation of cardiac patients. Therefore, systematic evaluation of the effects of different exercise intensities on the efficacy of cardiac function and quality of life in patients with CHF was done.

**Methods:**

Computer searches of PubMed, Web of Science, The Cochrane Library, Embase, SinoMed, the China National Knowledge Infrastructure, Wanfang, and VIP databases were conducted to collect randomized controlled trials of different exercise intensities applied to patients with CHF. Study selection and data extraction will be performed simultaneously by two independent reviewers, using the PEDro scale for quality assessment of the included literature. Publication bias will be assessed by funnel plot, and Begg and Egger tests. The *I*^2^ statistic and the chi-square (*χ*^2^) test will be used to assess heterogeneity. In addition, subgroup analyses will be performed for different left ventricular ejection fraction populations and different intervention cycles. All meta-analyses will be performed using Revman5.3 software.

**Results:**

The present study is a systematic review and meta-analysis program with no results. Data analysis will be completed after the program has been completed.

**Conclusion:**

This meta-analysis may provide more reliable, evidence-based evidence for the choice of exercise intensity in patients with CHF.

**Registration number:**

CRD42021276529

## 1 Introduction

There are 23 million patients with chronic heart failure (CHF) worldwide. It is increasing at a rate of 2 million per year.^[1]^ The 5-year mortality rate is much higher than most cancers.^[2]^ Heart failure is not a single pathological diagnosis but a clinical syndrome consisting of cardinal symptoms (e.g., breathlessness, ankle swelling, and fatigue). These symptoms result from structural and/or functional abnormalities of the heart, resulting in increased intracardiac pressure and insufficient cardiac output at rest or during exercise.^[3]^ One characteristic of CHF is exercise intolerance. Patients show reduced daily activities, prolonged sitting time, and decreased self-care ability, which negatively affects the activities required by patients in daily life, further reducing their independence and quality of life. The prognosis of heart failure has improved after using new drugs or cardiovascular implantable electronic device surgery. However, the mortality rate is still high.^[4]^ Regardless of the etiology, CHF can benefit from exercise-based cardiac rehabilitation (CR).^[5]^ A large body of research supports the use of exercise, such as high-intensity interval training, medium-intensity training, and resistance training, which can improve symptoms, quality of life, and physical function in patients with CHF.^[5]^

### 1.1 Literature review

Exercise training improves myocardial perfusion for patients with CHF by alleviating endothelial dysfunction, dilating coronary vessels, and stimulating new vessel formation through intermittent ischemia. Indeed, Santoso et al have demonstrated that aerobic exercise improves the N-terminal pro-B-type natriuretic peptide, ventilatory efficiency, aerobic capacity, maximal workload, and left ventricular function in patients with CHF.^[6]^ In addition, a meta-analysis showed that CHF patients could benefit more from resistance training programs.^[7]^ To date, several systematic reviews have examined the effects of different forms of exercise on patients with CHF. However, there is a lack of consensus on the optimal exercise intensity for the rehabilitation of patients with CHF.

### 1.2 Why it is essential to do this systematic review

Studies showed that exercise intensity is important in reversing left ventricular remodeling and improving aerobic capacity, endothelial function, and quality of life in cardiovascular disease.^[8]^ In patients with coronary artery disease, exercise training exercise load at the time of patient completion of CR was independently associated with prognosis. Since the 1980s, numerous studies have demonstrated the safety and effectiveness of moderate-intensity training.^[8,9]^ A recent study suggested that high-intensity interval training was superior to moderate-intensity training in patients with heart failure and restricted ejection fraction in altering left ventricular remodeling or aerobic capacity.^[10]^ However, Ellingsen et al^[11]^ found that high-intensity training could improve outcomes in three-week outpatient CR, but it may not benefit all patients. The relationship between exercise training load and clinical outcomes in patients with CHF is unclear. Therefore, this study combined relevant domestic and foreign literature and used meta-analysis to evaluate the effects of different exercise intensities on patients with CHF cardiac function and quality of life.

## 2 Methods

### 2.1 Search strategy and registration

**Figure 1:**
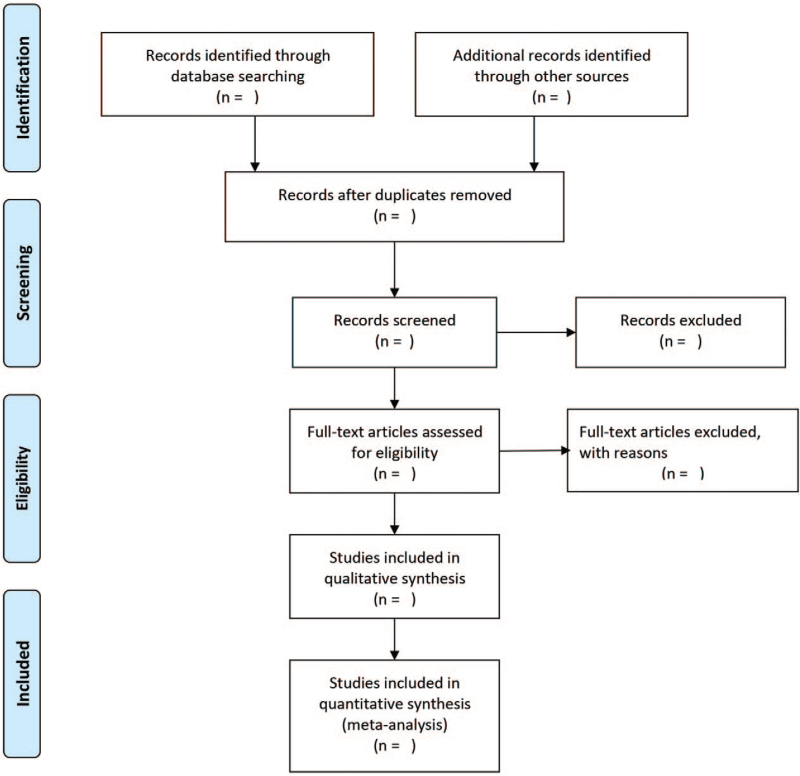
Flow diagram of study selection. This flow chart was used to depict the number of primary studies included and excluded in each stage of the study selection process.

The methods adopted for this review are compliant with the recommended Preferred Reporting Items for Systematic Review and Meta-Analysis checklist guidelines for systematic reviews.^[12]^ In addition, the Preferred Reporting Items for Systematic Review and Meta-Analysis flow diagram will be used to describe the number of primary studies that are included and excluded in each stage of the selection process (Fig. 1). The author will perform electronic searches of PubMed, Web of Science, The Cochrane Library, Embase, SinoMed, the China National Knowledge Infrastructure, Wanfang, and VIP databases and collected randomized controlled trials (RCTs) of different exercise intensities applied to patients with CHF. The search time limit is from establishing the database to March 2021. The syntax of this systematic review is a combination of Mesh terms and free text words. The details of the PubMed database search syntax are presented in Table 1. This protocol has been registered in the international prospective register of systematic reviews (PROSPERO; registration number: CRD42021276529).

**Table 1:** Search strategy for PubMed database.

Number	Strategy
#1	“Heart failure”[Mesh]
#2	“Heart decompensation”[Title/Abstract] OR “Cardiac failure”[Title/Abstract] OR “Right sided heart failure”[Title/Abstract] OR “Right-sided heart failure”[Title/Abstract] OR “Myocardial failure”[Title/Abstract] OR “Congestive heart failure”[Title/Abstract] OR “Left sided heart failure”[Title/Abstract] OR “Left-sided heart failure”[Title/Abstract] OR “Heart Failure”[Title/Abstract] OR “Chronic heart failure”[Title/Abstract]
#3	#1 OR #2
#4	“Exercise intensity”[Title/Abstract] OR “Exercise load”[Title/Abstract] OR “Sports dose”[Title/Abstract]
#5	#3 AND #4

### 2.2 Inclusion and exclusion criteria

#### 2.2.1 Types of literature

Studies will be screened for selection according to the review objectives and Participants, Interventions, Comparisons, Outcomes criteria. This systematic review and meta-analysis will include all randomized clinical trials involving moderate-intensity exercise intervention in the control group and high-intensity exercise in the experimental group. Exercise intensity is measured by self-perceived intensity grading (the Borg scale). The language will be limited to English and Chinese. We will exclude all republished documents and documents that provide insufficient information, incomplete data, inability to be included in the analysis, or unavailable full texts and documents with research design flaws.

#### 2.2.2 Types of patients

The inclusion criteria for participants will be as follows: (1) age ≥18 years; (2) diagnosed with CHF but with no limitation on gender, nationality, ethnicity. The exclusion criteria will be (1) patients with sudden onset of disease and unstable vital signs; (2) impaired mobility; (3) diagnosis of major depression, cognitive functioning disorder.

#### 2.2.3 Types of interventions and comparisons

Eligible studies will report the intensity of the patient's exercise, the duration, the frequency of the exercise, and the equipment used during the exercise. The target intensity of endurance exercise in heart failure patients is mainly based on peak oxygen consumption (PeakVO_2_) and metabolic equivalent. According to the grading standards for exercise intensity proposed by the 2020 European Society of Cardiology Guidelines on sports cardiology and exercise in patients with cardiovascular disease, if the anaerobic threshold is 50% to 70% PeakVO_2_ intensity is moderate, and for 70% PeakVO_2_ intensity is high intensity.^[13]^ The intensity of exercise training in the intervention group was high-intensity aerobic exercise with no restrictions on exercise form, frequency, and duration. Compared with the experimental group, the control group took moderate-intensity aerobic exercise outcomes.

### 2.3 Types of outcomes

The primary outcomes will be PeakVO_2_ and left ventricular ejection fraction (LVEF). PeakVO_2_ is an influential prognostic factor for heart failure.^[14]^ LVEF refers to the percentage of stroke volume to ventricular end-diastolic volume. It is one of the crucial indications for judging the type of heart failure.^[15]^ The secondary outcomes are quality of life. The Minnesota Living with Heart Failure Questionnaire or the Medical Outcomes Study item short-form health survey (SF-36) assesses the quality of life of patients.^[16]^

## 3 Data collection and analysis

### 3.1 Selection of studies

First, all articles that meet the search strategy were checked for duplicates, and after eliminating duplicates, the titles and abstracts of the remaining articles were carefully reviewed by two researchers (BH and FZ) to identify studies eligible for inclusion. Two researchers (BH and WQZ) will then independently evaluate the full text of potentially relevant articles. Conflicts will be resolved through discussion to reach a consensus. When consensus is not reached, a third researcher (BD) will act as an arbitrator.

#### 3.1.1 Data and information extraction

Two researchers (BH and FZ) independently screened the literature, extracted data, and cross-checked it. If there is a disagreement, it will be resolved through discussion or negotiation with the third researcher (BD). When selecting documents, read the title first, and after excluding irrelevant documents, read the abstract and full text to determine whether to include it.

A detailed data and information extraction form will be made by following: basic information (first author, publication year); participants’ characteristics (average age, gender, sample size); interventions (exercise frequency, time, duration of session, training cycles); comparisons (control mode); outcomes (PeakVO_2_, LVEF, quality of life questionnaire) (Table 2).

**Table 2:** Summary of the included RCTs.

Authors/publication year	Sample size (n = )	Age (years)	Sex	Intervention group	Control group	Outcome	Measurements
Author1	I: C:	I: C:	I: C:	Frequency & Duration: Duration of Session: Intensity (% of 1RM): Method of progression:	Frequency &Duration: Duration of Session: Intensity (% of 1RM): Method of progression:		

C = control group, I = intervention group, RCTs = randomized controlled trials.

#### 3.1.2 Dealing with missing data

We will contact the corresponding authors of studies by email to obtain data missing from published articles in case of insufficient information. However, if the authors do not respond to queries, we will calculate the missing data from other measures or estimate them from a similar study.

### 3.2 Assessment of risk of bias of included studies

The quality of the literature was independently evaluated by two researchers using the Physiotherapy Evidence Database (PEDro) scale (maximum score of 10).^[17]^ The PEDro scale is a dedicated research tool for evaluating the quality of RCTs in physiotherapy based on the Delphi inventory, designed to use the best evidence applied to enhance the effectiveness clinically and is increasingly being used in the field of rehabilitation. It includes an assessment of 11 aspects such as random allocation, inclusion conditions, allocation concealment, blinding, baseline characteristics, and outcome evaluation, with each entry scoring 1. Trial quality is defined using the PEDro scale: “good” 6 to 8 points, “fair” 4 to 5 points, “poor” ≤3 points and points are only awarded when a criterion is satisfied.

### 3.3 Assessment of heterogeneity

All analyses will be performed using Revman5.3 software on a personal laptop. Heterogeneity among primary studies will be evaluated by the *I*^2^ statistic and *χ*^2^ test as recommended by the Cochrane Handbook for Systematic Reviews of interventions.^[18]^ The guidelines are explained as follows: (1) 0 to 40% = no important heterogeneity; (2) 30% to 60% = moderate heterogeneity; (3) 50% to 90% = substantial heterogeneity; (4) 75% to 100% = considerable heterogeneity.

We will consider heterogeneity before data analysis. When there is significant heterogeneity in the studies (*I*^2^ > 50%), the results will be presented qualitatively in the text, not pool them. The use of random-effects models will be based on whether all included studies have a standard effect size and the results of statistical heterogeneity tests. When the *I*^2^ value is slightly above 50%, and there is an overlap between confidence interval (CI) and visual inspection in the forest plot, we will use a random-effects model for meta-analysis. We considered it statistically significant when *P* < .05.

### 3.4 Assessment of publication bias

When the number of included studies is ≥10, publication bias will be explored through funnel plots and tests of Begg and Egger. When the number of studies is <10, studies are used for analysis, we do not assess publication bias as the publication bias test produces unreliable results.

### 3.5 Data synthesis

#### 3.5.1 Descriptive analysis

First, a detailed reading of the full text of the included primary studies was performed, and information was extracted in two separate tables. The first table will provide the results of assessing the quality of the literature after evaluation using the PEDro scale. The second table will include the sample size of the study population, age, gender, intervention versus control group settings, and the outcome indicators measured.

We used RevMan5.3 for meta-analysis to synthesize included data. Weighted number differences (MD) were taken when the units of the measures were identical, and standard mean differences (SMD) were used for effect size analysis when the units were not identical, expressed as 95% CI, and differences were considered statistically significant at *P* < .05. Where inclusion in a study is inconsistent with the extraction of mean and standard deviation data, the relevant data will be transformed.^[19]^ Clinical heterogeneity was judged using the *χ*^2^ and *I*^2^ index; if *P* > .05 or *I*^2^ < 50% it indicates the absence of heterogeneity, a fixed-effect model was selected for effect size analysis; conversely, a random-effects model was selected for analysis. If statistical heterogeneity existed among the studies’ results, subgroup analysis and sensitivity analysis were further performed for possible sources of heterogeneity.

#### 3.5.2 Subgroup analysis

Subgroup analysis was performed on patients with different LVEF values at baseline. The subgroups were heart failure with preserved ejection fraction in the heart failure group with preserved ejection fraction: LVEF ≥ 50%; heart failure with reduced ejection fraction in the heart failure group with reduced ejection fraction: LVEF < 40%.^[3]^ In addition, subgroup analysis of different intervention cycles included in the literature, training cycles <12 weeks for one group and training cycles ≥12 weeks for one group, was performed to observe the effect of different intervention durations on outcome indicators.

#### 3.5.3 Sensitivity analysis

We will also implement a sensitivity analysis to explore the impact of methodological quality and sample size on the robustness of the review findings.

#### 3.5.4 Ethics and dissemination

Ethical approval is not required for this study as it is a systematic review protocol, and patients will not participate in this study.

## 4 Discussion

The choice of exercise intensity is a key aspect of cardiac rehabilitation in patients with CHF.^[20]^ Studies have found differences in the efficacy of training for patients at different exercise intensities, with a tendency to benefit more as exercise intensity increases. High-intensity exercise can effectively improve systolic cardiac function and accelerate oxygen delivery and utilization in skeletal muscle or frontal lobes in a short period, in terms of central hemodynamic adaptation.^[21]^ At the same time, high-intensity exercise enhances skeletal muscle blood flow, accelerates the clearance of exercise-generated metabolites, and improves exercise hyperventilation, thus increasing exercise tolerance plays an improvement role in patients’ LVEF and PeakVO_2_.^[22]^

However, patients are often required to cooperate with high-intensity exercise for a short period during high-intensity exercise rehabilitation, resulting in poor exercise experience, decreased comfort, and increased negative emotions, which do not motivate patients’ treatment and compliance and eventually lead to patients’ reluctance to cooperate with treatment. Then, whether high-intensity exercise is suitable for rehabilitation exercise for CHF patients is still debatable. Therefore, this systematic review conducted a meta-analysis on the efficacy of different intensity exercises on patients’ cardiac function and patients’ quality of life improvement and analyzed the benefit of different exercise intensities on different ejection fraction populations from different LVEF populations. The results of the short- or long-term benefits of different exercise intensities on patients were explored in terms of the intervention period. The article aims to provide a reference for clinical medical decision-makers in selecting the appropriate exercise intensity for their patients.

## Author contributions

YB developed the search strategy and drafted the protocol, and revised by BD and BH; BH, FZ, and WQZ independently worked on study selection, quality assessment, and data extraction; YB worked on data synthesis; BD resolved any divergences.

**Conceptualization:** Yan Bai, Bing Deng.

**Data curation:** Fan Zhang, Wenqin Zhou.

**Formal analysis:** Bin Hua, Bing Deng.

**Funding acquisition:** Bing Deng.

**Methodology:** Fan Zhang.

**Project administration:** Wenqin Zhou.

**Resources:** Wenqin Zhou.

**Software:** Fan Zhang.

**Supervision:** Bing Deng.

**Writing – original draft:** Yan Bai, Bin Hua.
